# Global and regional estimates of the contribution of herpes simplex virus type 2 infection to HIV incidence: a population attributable fraction analysis using published epidemiological data

**DOI:** 10.1016/S1473-3099(19)30470-0

**Published:** 2020-02

**Authors:** Katharine J Looker, Nicky J Welton, Keith M Sabin, Shona Dalal, Peter Vickerman, Katherine M E Turner, Marie-Claude Boily, Sami L Gottlieb

**Affiliations:** a Population Health Sciences, Bristol Medical School, University of Bristol, Bristol, UK; b Bristol Veterinary School, University of Bristol, Bristol, UK; c UNAIDS, Geneva, Switzerland; d Department of HIV/AIDS, WHO, Geneva, Switzerland; e Department of Reproductive Health and Research, WHO, Geneva, Switzerland; f MRC Centre for Global Infectious Disease Analysis, Department of Infectious Disease Epidemiology, Imperial College London, London, UK

## Abstract

**Background:**

A 2017 systematic review and meta-analysis of 55 prospective studies found the adjusted risk of HIV acquisition to be at least tripled in individuals with herpes simplex virus type 2 (HSV-2) infection. We aimed to assess the potential contribution of HSV-2 infection to HIV incidence, given an effect of HSV-2 on HIV acquisition.

**Methods:**

We used a classic epidemiological formula to estimate the global and regional (WHO regional) population attributable fraction (PAF) and number of incident HIV infections attributable to HSV-2 infection by age (15–24 years, 25–49 years, and 15–49 years), sex, and timing of HSV-2 infection (established *vs* recently acquired). Estimates were calculated by incorporating HSV-2 and HIV infection data with pooled relative risk (RR) estimates for the effect of HSV-2 infection on HIV acquisition from a systematic review and meta-analysis. Because HSV-2 and HIV have shared sexual and other risk factors, in addition to HSV-related biological factors that increase HIV risk, we only used RR estimates that were adjusted for potential confounders.

**Findings:**

An estimated 420 000 (95% uncertainty interval 317 000–546 000; PAF 29·6% [22·9–37·1]) of 1·4 million sexually acquired incident HIV infections in individuals aged 15–49 years in 2016 were attributable to HSV-2 infection. The contribution of HSV-2 to HIV was largest for the WHO African region (PAF 37·1% [28·7–46·3]), women (34·8% [23·5–45·0]), individuals aged 25–49 years (32·4% [25·4–40·2]), and established HSV-2 infection (26·8% [19·7–34·5]).

**Interpretation:**

A large burden of HIV is likely to be attributable to HSV-2 infection, even if the effect of HSV-2 infection on HIV had been imperfectly measured in studies providing adjusted RR estimates, potentially because of residual confounding. The contribution is likely to be greatest in areas where HSV-2 is highly prevalent, particularly Africa. New preventive interventions against HSV-2 infection could not only improve the quality of life of millions of people by reducing the prevalence of herpetic genital ulcer disease, but could also have an additional, indirect effect on HIV transmission.

**Funding:**

WHO.

## Introduction

An estimated 417 million people globally had herpes simplex virus type 2 (HSV-2) infection in 2012,^1^ a lifelong infection that causes genital herpes.^2^ Prevalence of HSV-2 infection is highest in the WHO African region (32% *vs* 11% globally),^1^ which also has the highest HIV burden. According to UNAIDS estimates, in 2016, there were an estimated 25·4 million people living with HIV in the WHO African region, of a total of 36·3 million people living with HIV globally. Of the estimated 1·5 million incident HIV infections among individuals aged 15–49 years in 2016, 950 000 (61%) were in the WHO African region. HSV-2 and HIV infections are closely associated; in the HIV Prevention Trials Network 071 study in southern Africa, HIV prevalence was 41% in individuals with HSV-2 infection compared with 6% in those without HSV-2 infection.^3^

Evidence suggests there is a strong biological association between HIV and HSV-2 infections—specifically, that HSV-2 infection increases susceptibility to HIV acquisition, both viruses increase transmissibility of the other, and HSV-2 disease can become severe among people with HIV-related immunosuppression.^4^, ^5^ The best characterised biological association (cofactor effect) between HSV-2 and HIV is the effect of HSV-2 infection on HIV acquisition. A 2017 systematic review and meta-analysis of 55 prospective studies reported that the risk of HIV acquisition was almost tripled in the presence of established (prevalent) HSV-2 infection and quintupled in the presence of recently acquired (incident) HSV-2 infection.^6^ The biological plausibility for a cofactor effect of HSV-2 infection on HIV acquisition is compelling: ulcerative first episodes and recurrences of HSV-2 infection result in disruption to the epithelial barrier, which facilitates the entry of HIV. The recruitment of immune cells to control initial HSV-2 infection and frequent reactivations (viral replication and shedding from the genital area) concentrates target cells for HIV in the genital area.^4^, ^5^, ^7^ Immune cells are recruited to the genital area regardless of whether viral reactivation is accompanied by symptoms,^8^ and this effect is seen even after 2 months of valaciclovir therapy.^5^, ^9^ Biological data are consistent with the finding that HIV acquisition risk is higher for individuals recently infected with HSV-2 than for those with established infection,^6^ as the first (symptomatic) episode of infection is longer in duration and more severe than subsequent recurrences^10^, ^11^ and the number of days with recurrences and viral shedding (and thus with mucosal inflammation) is highest in the first year.^5^, ^12^, ^13^

**Research in context**

**Evidence before this study**

Biological and epidemiological evidence suggests that herpes simplex virus type 2 (HSV-2) infection increases the subsequent risk of acquiring HIV infection. A 2017 systematic review and meta-analysis of 55 prospective studies in which HSV-2 infection was known to precede HIV infection found the risk of HIV acquisition, adjusted for other shared risk factors for HIV and HSV-2 infections, to be almost tripled with established HSV-2 infection (acquired >1 year ago) and quintupled in the presence of recently acquired HSV-2 infection (acquired within the past year). Although published estimates exist of the population attributable fraction (PAF) of incident HIV infection attributable to HSV-2 infection (as identified through a scoping review done for this study), they are available only for certain African settings. For the scoping review we searched PubMed from database inception to May, 2019, using search terms relating to HIV, HSV-2, and PAF.

**Added value of this study**

We combined the most recently available data on the burden of HSV-2 and HIV with pooled adjusted relative risk (RR) estimates for the effect of HSV-2 infection on HIV acquisition and used a classic epidemiological formula to translate these data into percentages and numbers of incident HIV infections attributable to HSV-2 infection in 2016. In so doing, we produced the first global and WHO-regional estimates of the potential contribution of HSV-2 infection to the HIV epidemic. These estimates are needed for improving our understanding of the extent to which HSV-2 infection contributes to HIV infections and how this varies across populations, which in turn informs where future intervention efforts would be best targeted to have maximum impact.

**Implications of all the available evidence**

HSV-2 infection is an important risk factor for HIV acquisition, and people with known HSV-2 infection could benefit from heightened HIV prevention efforts. We used only adjusted RR estimates in our PAF calculations to most closely reflect biological rather than behavioural risk. Although residual confounding could still have been present, our findings suggest that the contribution of HSV-2 infection to HIV acquisition is substantial given the high prevalence of HSV-2 infection globally, even if the effect of HSV-2 infection on HIV transmission is not necessarily measured perfectly in the available studies. Interventions targeted against HSV-2, such as new vaccines or microbicides, have the potential to improve the lives of millions of people by reducing recurrent genital herpetic symptoms. In addition, in the presence of an effect of HSV-2 infection on HIV acquisition, prevention measures against HSV-2 transmission could have a further, indirect benefit on HIV.

A few studies have estimated the percentage of incident HIV infections that are attributable to HSV-2 infection, known as the population attributable fraction (PAF).^14^, ^15^, ^16^, ^17^, ^18^, ^19^, ^20^ Existing estimates are, to our knowledge, only available for some settings in the WHO African region.^15^, ^16^, ^17^, ^18^, ^19^, ^20^ Broader PAF estimation would enable a preliminary understanding of the potential global contribution of HSV-2 to HIV infection and how this contribution might differ across populations according to epidemic setting (ie, prevalence and incidence of HSV-2 and incidence of HIV). In this Article, we present the first global and regional (WHO regional) estimates of the PAF and number of incident HIV infections attributable to HSV-2 infection by age, sex, and timing of HSV-2 infection (established *vs* recently acquired). Estimates are provided for the general population and separately for female sex workers (FSWs) and men who have sex with men (MSM).

## Methods

### PAF formula and data sources

Our global and WHO regional (Americas, African, Eastern Mediterranean, European, South-East Asian, and Western Pacific) PAF estimates, were derived using the classic (Levin) epidemiological formula for polytomous exposure,^14^ which accounts for recently acquired and established HSV-2 infection:

$$PAF(\%)=\frac{\left[P_{HSV-2\_established}\times (RR_{HSV-2\_established}-1)+P_{HSV-2\_recent}\times (RR_{HSV-2\_recent}-1)\right]}{\left[1+P_{HSV-2\_established}\times (RR_{HSV-2\_established}-1)+P_{HSV-2\_recent}\times (RR_{HSV-2\_recent}-1)\right]}\times 100\%$$

where *P*_HSV-2_established_ is the proportion of individuals with established HSV-2 infection in the overall population (ie, individuals infected and uninfected with HSV-2), *P*_HSV-2_recent_ is the proportion of individuals with recently acquired HSV-2 infection in the overall population (ie, individuals infected and uninfected with HSV-2), *RR*_HSV-2_established_ is the relative risk (RR) of HIV acquisition among individuals with established HSV-2 infection versus those without HSV-2 infection, and *RR*_HSV-2_recent_ is the RR of HIV acquisition among individuals with recently acquired HSV-2 infection versus those without HSV-2 infection.

Recently acquired HSV-2 infection was defined as infection acquired within the past year and established HSV-2 infection as an HSV-2 infection acquired more than 1 year ago.

Once the PAF has been calculated, it can then be applied to the total number of incident HIV infections from sexual transmission in a given population for a given year to obtain the number of incident HIV infections attributable to HSV-2 infection in that population for that year.

Data on the RR of HIV acquisition, occurrence of established or recently acquired HSV-2 infection, and HIV incidence to inform parameter values in the PAF formula were taken from the most recent literature reviews^1^, ^6^ or were official UNAIDS estimates available at the time of analysis, as specified in detail in the next paragraphs. Data sources for the parameters had slightly varying time frames but were applied to 2016 UNAIDS HIV incidence estimates to estimate the number of incident HIV infections in 2016 that were attributable to HSV-2 infection.

We extracted pooled RR estimates for the effect of HSV-2 infection on HIV acquisition (combining hazard ratios, rate ratios, and odds ratios across prospective studies) and 95% CIs from our 2017 systematic review and meta-analysis of 55 prospective studies from literature searched between Jan 1, 2003, and May 25, 2017, and from studies published before 2003 identified in a 2006 systematic review.^6^, ^21^ To minimise confounding from shared risk factors for HIV and HSV-2 infections and any mutual effect of HIV infection on HSV-2 acquisition, RR estimates were based on studies that adjusted for shared risk factors and in which HSV-2 infection was known to have preceded HIV acquisition. Table 1 summarises estimates and number of studies available by sex, risk population (general population, MSM, FSWs, and other high-risk groups), and time since HSV-2 infection (recently acquired or established).^6^ All studies included in the meta-analysis for the general population, and all but one study of FSWs, were from Africa, whereas all studies of MSM were from outside Africa (mostly the USA, but also Australia, China, Thailand, and the Caribbean). Only one study reported RR estimates for the effect of recently acquired HSV-2 infection on HIV acquisition in men.

**Table 1:** Studies informing pooled, adjusted RR estimates of HIV acquisition attributable to HSV-2 infection by time since HSV-2 infection, risk population, and sex^6^

	**Established HSV-2 infection**			**Recently acquired HSV-2 infection**		
	Number of studies informing RR estimate	Pooled adjusted RR (95% CI)	*I^2^*	Number of studies informing RR estimate	Pooled adjusted RR (95% CI)	*I^2^*
General population*	22	2·7 (2·2–3·4)	59%	6	4·7 (2·2–10·1)	64%
Women only	11	2·5 (1·8–3·4)	68%	5	7·2 (4·5–11·5)	0%
Men only	10	3·1 (2·2–4·3)	48%	1	1·1 (0·4–3·1)†	..
Both sexes	1	8·7 (1·1–67·2)	..	..	..	..
Men who have sex with men†	7	1·7 (1·4–2·1)	26%	1	2·8 (0·8–9·9)†	..
Female sex workers‡	7	1·5 (0·8–2·7)	65%	1	3·0 (1·6–5·3)†	..
Other high-risk groups (women, men, or both)§¶	11	Pooling not done‖	..	..	..	..

HSV=herpes simplex virus. RR=relative risk.

* All studies were in Africa.

† Four studies informing the RR estimate for established HSV-2 infection were in the Americas, one in South-East Asia, one in Western Pacific, and one in more than one WHO region; the one study informing the RR estimate for recently acquired HSV-2 infection was in the Americas.

‡ Six studies informing the RR estimate for established HSV-2 infection were in Africa and one was in South-East Asia; the one study informing the RR estimate for recently acquired HSV-2 infection was in Africa.

§ High-risk populations were female workers in bars, hotels, and food and recreational facilities; serodiscordant couples; male trucking-company employees; male military conscripts; attendees of sexually transmitted infection clinics; and women reported as being at high risk.

¶ Six studies informing the RR estimate for established HSV-2 infection were in the Americas, four were in South-East Asia, and one was in Western Pacific.

‖ Range of individual-study estimates was 0·5 (95% CI 0·2–1·1) to 4·3 (1·5–12·4).

Estimates of the proportion of individuals with established HSV-2 infection (with 95% uncertainty intervals [UIs]) or recently acquired HSV-2 infection (without 95% UIs) for the general population, stratified by age (in 5-year age bands from age 15 years to 49 years), sex, and WHO region, were available from the 2012 WHO estimates for prevalent and incident HSV-2 infection, which were informed by a comprehensive literature review up to February, 2014, that included 111 studies.^1^ For those estimates, incidence was derived by calibrating the force of infection (incidence per susceptible individual) to prevalence, assuming a constant force of infection by age.^22^ Prevalence was calculated for each year of age, and the force of infection was applied to the number of susceptible individuals at that age to estimate the number of new infections over 1 accumulated year of age.

In addition, we combined age-stratified data from 44 studies of HSV-2 prevalence among MSM and FSWs (excluding those with HIV) with year of data collection 2000 or later and a sample size of 20 or greater. These data were collated during the same literature review but not used in the 2012 WHO estimates (appendix 1).^1^ After adjusting for assay performance, we weighted the data by sample size using a random-effects model to derive pooled estimates of the proportion of MSM or FSWs with established HSV-2 infection by WHO region. As HSV-2 prevalence data among MSM were not available for the African and Eastern Mediterranean regions, we estimated the proportion of MSM with established HSV-2 infection in these regions by multiplying the proportion of men in the general populations in those regions with established HSV-2 infection by the global (ie, all other WHO regions) mean ratio of established HSV-2 infection in MSM to established HSV-2 infection in the general male population. For example, for African MSM:

$$P_{HSV-2\_established\_r=5,MSM}=\left[\left(\sum_{r=1}^{r=4}\frac{P_{HSV-2\_established\_r,MSM}}{P_{HSV-2\_established\_r,k=1}}\right)/4\right]\times P_{HSV-2\_established\_r=5,k=1}$$

where *r* denotes region (*r*=1 for the Americas, *r*=2 for European, *r*=3 for South-East Asian, *r*=4 for Western Pacific, *r*=5 for African, and *r*=6 for Eastern Mediterranean) and *k* denotes sex (*k*=0 for women and *k*=1 for men). We calculated the global mean ratio of established HSV-2 infection in MSM to established HSV-2 infection in the general male population,

$$\left[\left(\sum_{r=1}^{r=4}\frac{P_{HSV-2\_established\_r,MSM}}{P_{HSV-2\_established\_r,k=1}}\right)/4\right]$$

to be 2·8.

We used UNAIDS estimates of the annual number of incident HIV infections by WHO region, age (*a*; 15–24 years, 25–49 years, and 15–49 years), and sex for 2016 (*N*_HIV_r,a,k_). Not all estimates had 95% UIs so we derived them when necessary on the basis of the size of available uncertainty bounds for other estimates (as a proportion of the estimate). In addition, we used UNAIDS estimates of the distribution (relative proportion, *X*) of all incident HIV infections by key population (MSM, FSWs, people who inject drugs [PWID], transgender individuals, sexual partners of high-risk individuals, and other [all remaining individuals]) that were calculated by WHO region for 2015.^23^ We used *X* to estimate the number of incident HIV infections occurring in FSWs (*N*_HIV_r,FSW_), MSM (*N*_HIV_r,MSM_), and PWID (*N*_HIV_r,PWID_) in each region, assuming for PWID equal proportions in women and men and that all HIV transmissions were through contaminated equipment:

$$N_{HIV\_r,FSW}=\sum_{a,k}N_{HIV\_r,a,k}\times X_{r,FSW}$$

$$N_{HIV\_r,MSM}=\sum_{a,k}N_{HIV\_r,a,k}\times X_{r,MSM}$$

$$N_{HIV\_r,a,k,PWID}=N_{HIV\_r,a,k}\times X_{r,PWID}$$

The number of incident infections among PWID was then used to estimate the number of incident HIV infections due to sexual transmission alone: *N*_HIV_r,a,k_sexual_ = *N*_HIV_r,a,k_ – *N*_HIV_r,a,k_PWID_. The PAF of incident HIV attributable to HSV-2 infection was calculated both for incident HIV infection from sexual transmission only (since HSV-2-associated HIV infection is sexually acquired) and for all incident HIV infections (with HIV infections in PWID being unaffected by HSV-2). The PAF for all incident HIV infections was calculated by multiplying the PAF for incident HIV infections from sexual transmission only by *N*_HIV_r,a,k_sexual_/*N*_HIV_r,a,k_.

### PAF estimation

We derived global and regional (WHO regional) estimates of the PAF and number of incident HIV infections attributable to HSV-2 infection for the general population by age (15–24 years, 25–49 years, and 15–49 years), sex, and timing of HSV-2 infection, and separately for MSM and FSWs. The age range 15–49 years and two age groups within this range were chosen because HSV-2 infection and HIV incidence estimates were available for individuals aged 15–49 years and for the age groups 15–24 years and 25–49 years. The age range 15–49 years also reflects peak exposure to HSV-2 and HIV. All available estimates and assumptions made to derive the PAF are summarised in appendix 2 (pp 6–8).

We used RR estimates in the general population by sex in the PAF calculations for women, men, and women and men combined. We used the RR for the effect of recently acquired HSV-2 infection on HIV acquisition in men and women combined in the PAF formula for men because only one study informed the RR for the effect in men (table 1). We used specific RR estimates for MSM and FSWs in the PAF calculations for these two key population groups. We did not estimate the PAF for the contribution of recently acquired HSV-2 infection to HIV incidence in MSM and FSWs given the scarcity of data to inform the proportion of individuals with recently acquired HSV-2 infection.

The global number of incident HIV infections attributable to HSV-2 infection was obtained by summing the numbers for all WHO regions. Global PAF estimates were then derived from the total number of incident HIV infections attributable to HSV-2 infection.

We derived 95% UIs of PAF estimates and estimates of the number of incident HIV infections attributable to HSV-2 infection using a Monte Carlo sampling method to sample the proportion of individuals with established HSV-2 infection, RR estimates, and HIV incidence (*P*_HSV-2_established_, *RR*_HSV-2_established_, *RR*_HSV-2_recent_, *N*_HIV_) within plausible ranges. We ran the Monte Carlo sampling 1000 times in Microsoft Excel 2003 (and above). *P*_HSV-2_established_ was sampled in two steps: first, sampling on the log-odds scale from a normal distribution, with the mean equal to the log-odds of infection and the SD equal to the SE of the log-odds of infection, and then back-transforming from the log-odds scale to a probability to use in the PAF calculations. *RR*_HSV-2_established_ and *RR*_HSV-2_recent_ were sampled using the same two steps as for *P*_HSV-2_established_ but on the log scale rather than the log-odds scale. *N*_HIV_ was sampled from a normal distribution with the mean equal to the number of infections and the SD equal to the SE of the number of infections, but sampled values were truncated at zero. We did not sample the proportion of individuals with recently-acquired HSV-2 infection, since 95% UIs were unavailable.^1^ The 95% UI was based on the 2·5 and 97·5 percentiles from the set of 1000 generated estimates.

All calculations were done with Microsoft Excel. A completed GATHER checklist is given in appendix 2 (p 5).

### Role of the funding source

The funder of the study had no role in study design, data collation, data analysis, data interpretation, or writing of the report. However, SLG, affiliated with WHO, commissioned the study, contributed to the direction of the work, and edited and commented on the drafts. The corresponding author had full access to all the data in the study and had final responsibility for the decision to submit for publication.

## Results

We estimated that the global PAF of incident HIV infection from sexual transmission in 2016 that was attributable to HSV-2 infection was 29·6% (95% UI 22·9–37·1). The PAF was highest for the WHO African region (37·1%, 95% UI 28·7–46·3) and the Americas (21·3%, 14·7–29·4), and was 11–13% elsewhere in the world (figure; table 2).

**Figure:**
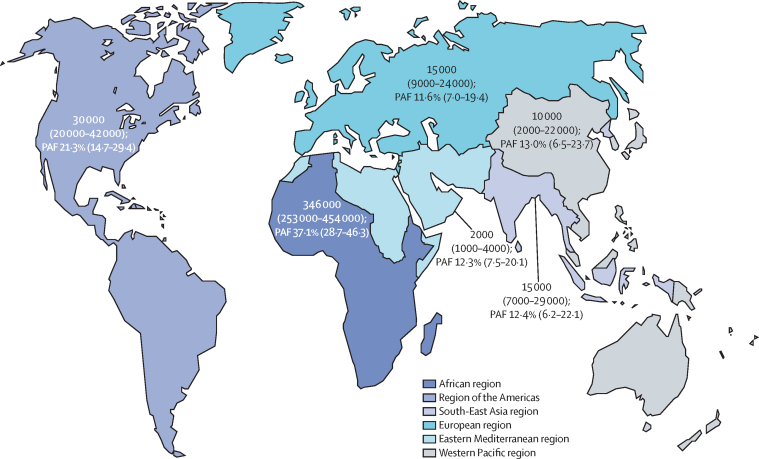
Estimated number and PAF of incident HIV infections among individuals aged 15–49 years in 2016 that were attributable to HSV-2 infection, by WHO region Data are n (95% UI) or % (95% UI). Number of incident HIV infections attributable to HSV-2 infection is given to the nearest thousand. 95% UIs were generated through separate sampling for each WHO region and globally; therefore, regional lower and upper bounds do not sum to the global bounds. PAF=population attributable fraction. UI=uncertainty interval.

**Table 2:** Estimated PAF and number of incident HIV infections in 2016 attributable to HSV-2 infection among individuals aged 15–49 years, overall and by WHO region

	**PAF of incident HIV infection attributable to HSV-2 infection**				**Number of incident HIV infections attributable to all HSV-2 infections**†	**Number of incident HIV infections from sexual transmission**†‡	**Number of incident HIV infections from all routes of HIV transmission**†§
	All HSV-2 infections	Established HSV-2 infections	Recently acquired HSV-2 infections	All incident HIV infection*			
African	37·1% (28·7–46·3)	33·7% (24·8–43·1)	3·4% (1·3–7·5)	36·4% (28·1–45·4)	346 000 (253 000–454 000)	932 000	951 000
Americas	21·3% (14·7–29·4)	19·2% (12·4–27·5)	2·1% (0·8–4·6)	20·5% (14·1–28·2)	30 000 (20 000–42 000)	140 000	146 000
Eastern Mediterranean	12·3% (7·5–20·1)	11·5% (6·6–19·5)	0·8% (0·3–1·9)	5·9% (3·6–9·7)	2000 (1000–4000)	16 000	33 000
European	11·6% (7·0–19·4)	10·6% (5·9–18·3)	1·0% (0·4–2·2)	7·4% (4·5–12·4)	15 000 (9000–24 000)	126 000	198 000
South-East Asian	12·4% (6·2–22·1)	11·2% (5·1–21·2)	1·2% (0·4–2·6)	11·5% (5·8–20·5)	15 000 (7000–29 000)	124 000	134 000
Western Pacific	13·0% (6·5–23·7)	11·8% (5·2–22·5)	1·2% (0·4–2·6)	11·0% (5·5–20·1)	10 000 (2000–22 000)	74 000	87 000
Global average (all regions)	29·6% (22·9–37·1)	26·8% (19·7–34·5)	2·7% (1·0–6·0)	27·0% (20·8–33·9)	420 000 (317 000–546 000)	1 411 000	1 548 000

Data are % (95% UI), n (95% UI), or n. PAF=population attributable fraction. UI=uncertainty interval.

* Includes incident HIV infections from all routes of transmission (not just sexual transmission).

† Estimates are shown to the nearest thousand; they are based on 2016 HIV incidence data, 2012 HSV-2 infection estimates,^1^ RR estimates from a review of literature published up until 2017,^6^ and key population breakdown of HIV incidence for 2015.^23^

‡ Denominators for calculation of PAFs of incident HIV infection via sexual transmission attributable to HSV-2 infection.

§ Denominators for calculation of PAFs of incident HIV infection via all routes of transmission attributable to HSV-2 infection.

Of the estimated 1·4 million incident HIV infections from sexual transmission among individuals aged 15–49 years globally in 2016, we estimated that 420 000 (95% UI 317 000–546 000) were attributable to HSV-2 infection (table 2). Most incident HIV infections attributable to HSV-2 infection were in the WHO African region (figure; table 2).

The PAF and number of incident HIV infections attributable to HSV-2 infection in individuals aged 15–49 years were higher among women than men (table 3), reflecting the higher proportion of women than men with HSV-2 infection and the higher RR for women than for men for the effect of recently acquired HSV-2 infection on HIV acquisition (table 1).^6^ For women and men combined, the PAF was higher for individuals aged 25–49 years than for those aged 15–24 years (table 3). Similar patterns by age and sex were seen by region (appendix 2 pp 9, 10).

**Table 3:** Estimated global PAF and number of incident HIV infections in 2016 attributable to HSV-2 infection, by age, sex, and risk population

		**PAF of incident HIV infections from sexual transmission attributable to HSV-2 infection**			Number of incident HIV infections attributable to all HSV-2 infections*	Number of incident HIV infections from sexual transmission*†
		All HSV-2 infections	Established HSV-2 infections	Recently acquired HSV-2 infections		
**General population**						
Women						
	15–24 years	30·8% (22·3–40·2)	21·4% (11·8–31·7)	9·4% (5·2–15·6)	105 000 (61 000–156 000)	340 000
	25–49 years	36·4% (24·7–46·9)	33·7% (21·1–44·8)	2·7% (1·5–4·8)	127 000 (76 000–182 000)	348 000
	15–49 years	34·8% (23·5–45·0)	29·6% (17·2–40·6)	5·1% (2·8–8·9)	239 000 (140 000–357 000)	689 000
Men						
	15–24 years	17·9% (11·0–27·1)	14·6% (8·1–23·6)	3·3% (0·9–7·5)	41 000 (20 000–72 000)	231 000
	25–49 years	30·2% (19·6–40·9)	28·4% (17·7–39·2)	1·8% (0·5–4·1)	148 000 (76 000–245 000)	492 000
	15–49 years	26·2% (16·9–37·3)	23·9% (14·6–34·7)	2·3% (0·7–5·3)	189 000 (99 000–314 000)	723 000
Women and men combined						
	15–24 years	23·2% (16·8–31·0)	18·6% (12·8–25·4)	4·6% (1·7–10·1)	133 000 (94 000–181 000)	572 000
	25–49 years	32·4% (25·4–40·2)	30·6% (23·3–38·5)	1·8% (0·7–4·1)	272 000 (207 000–347 000)	840 000
**Key risk populations**‡						
Female sex workers		26·7% (0·0–56·5)	26·7% (0·0–56·5)	..	20 000 (0–44 000)§	77 000
Men who have sex with men		19·9% (8·7–28·7)	19·9% (8·7–28·7)	..	40 000 (17 000–62 000)§	203 000

Data are % (95% UI), n (95% UI), or n. The number of incident HIV infections attributable to HSV-2 infection was calculated for each age and sex group separately; therefore, estimates do not sum exactly across rows. PAF=population attributable fraction. UI=uncertainty interval.

* Estimates are shown to the nearest thousand; they are based on 2016 HIV incidence data, 2012 HSV-2 infection estimates,^1^ key population HSV-2 prevalence data reviewed as part of the 2012 work on HSV-2 infection estimates,^1^ RR estimates from a review of literature published up until 2017,^6^ and key population breakdown of HIV incidence for 2015.^23^

† Denominators for calculation of PAFs.

‡ Only established HSV-2 infections were considered, and not recently acquired ones.

§ These numbers form a subset of the global numbers and are not in addition to them.

We estimated that 20 000 (0–44 000) incident HIV infections in FSWs and 40 000 (17 000–62 000) incident HIV infections in MSM in 2016 were attributable to established HSV-2 infection (table 3).

The PAF of incident HIV infection attributable to recently acquired HSV-2 infection was consistently low across regions (less than 4%; table 2), but it had a larger contribution among younger age groups and women (table 3).

The PAF of incident HIV infection acquired from all transmission routes attributable to HSV-2 infection was similar to that of incident HIV infection attributable to HSV-2 infection acquired from sexual transmission only (table 3). The exception was for regions where a substantial proportion of HIV infections are acquired in PWID, notably the Eastern Mediterranean (33 000 incident infections from all routes of transmission *vs* 16 000 incident infections from sexual transmission) and European regions (198 000 *vs* 126 000).^23^

## Discussion

We estimated that 420 000 (95% UI 317 000–546 000; PAF 29·6%, 95% UI 22·9–37·1) of 1·4 million incident HIV infections acquired via sexual transmission in individuals aged 15–49 years in 2016 were attributable to HSV-2 infection. However, this global figure masks large regional differences: by far the largest number and proportion of incident HIV infections attributable to HSV-2 infection were in Africa. This finding reflects the larger burden of HSV-2 and HIV infections in this region than elsewhere. The estimated PAF was highest for women and individuals aged 25–49 years because of a greater proportion of people in these populations having HSV-2 infection.^1^ It was also higher for established than recently acquired HSV-2 infection, despite the higher RR for recently acquired than for established HSV-2 infection (table 1); this finding can be explained by many more people having established than recently acquired HSV-2 infection, especially at older ages. The PAF was estimated to be lower for FSWs and MSM than for the general population because of lower RR estimates for these key risk populations.

To our knowledge, we report the first global and regional estimates of the PAF and number of incident HIV infections attributable to HSV-2 infection. These estimates are useful for understanding the potential magnitude of the contribution of HSV-2 infection to HIV, which can help stimulate development of new interventions and guide where future prevention efforts would be best targeted for optimal effect. Our PAF estimates were based on the most recent WHO estimates of HSV-2 prevalence and incidence among general populations (informed by 111 studies), and HSV-2 prevalence estimates for FSWs and MSM (based on 44 studies) were from a global review.^1^ The estimates were also based on RR estimates of the effect of HSV-2 infection on HIV acquisition from a 2017 systematic review and meta-analysis^6^ of 55 prospective studies and the most recently available UNAIDS estimates of HIV incidence and breakdown by key populations.^23^ However, the PAF estimates have some limitations. First, the same limitations and caveats affecting the estimates of HSV-2 infection^1^ and HIV infection^23^ apply here, such as the availability and generalisability of contributing studies. The HSV-2 prevalence and incidence estimates used in our PAF calculations were based on 2012 population data. However, they used prevalence data published up to 2014 and overall prevalence changes slowly since HSV-2 is a life-long infection. We were also limited by the number and types of studies contributing data to the RR estimates, which did not allow use of age-specific, sex-specific, and region-specific RR estimates in all calculations and required us to make additional assumptions.^6^ For example, we applied the RR for recently acquired HSV-2 infection among women and men combined to calculate PAF estimates for men in the general population because only one estimate was available for men. Similarly, we did not use RR estimates stratified by age or WHO region because of the small number of studies that were available. In the systematic review,^6^ there was some evidence that, for recently acquired HSV-2 infection, RR estimates were higher for younger individuals than older ones. However a univariate meta-regression analysis (combining RR estimates for established and recently acquired HSV-2 infection) did not find a significant association for age on RR estimates.^6^ In the systematic review,^6^ it was not possible to separate the effects of region and risk group because there were no RR estimates for general populations outside of Africa. However, RR estimates for established HSV-2 infection, which contributed more to HIV incidence than recently acquired infection, were the best informed by data and suggested similar estimates among men and women and by age. RR estimates might be different for general populations outside of Africa but without any data it is difficult to speculate on this possibility.

The issues about underlying data availability and assumptions were partly mitigated by calculating UIs. We were unable to account for uncertainty in the proportion of individuals with recently acquired HSV-2 infection, which might, in principle, have led to underestimation of uncertainty in the PAF estimates. However, this underestimation is unlikely because, as noted above, individuals with recently acquired HSV-2 infection are a minority and as a result contributed little to the PAF estimates.

A second limitation of the PAF estimates is that, by definition, they assume a causal link—in this case, an association between HSV-2 and HIV that is not solely a reflection of confounding.^24^ Even with the most rigorously collected and analysed observational data, it is difficult to completely disentangle the biological cofactor effect from other dynamics (eg, confounding). In deriving the RR estimates applied here,^6^ only adjusted estimates were pooled, the majority of which were adjusted for sexual behaviour. In addition, adjustment was found not to markedly affect the magnitude of the RR estimates, and RR estimates were also remarkably consistent across studies.^6^ Furthermore, we restricted our estimates of HIV acquisition attributable to recently acquired HSV-2 infection to RRs from studies with known timing of HSV-2 infection in relation to HIV. We found that recently acquired HSV-2 infection had a stronger cofactor effect on HIV acquisition than established HSV-2 infection,^6^ which could favour a biological explanation since a first episode of HSV-2 infection is more severe than subsequent recurrences.^10^, ^12^, ^25^ However, finding of an association between HSV-2 infection and HIV acquisition and biological plausibility do not eliminate the possibility of residual confounding of the observed association between HSV-2 and HIV infections, especially in relation to sexual network and partner characteristics.^24^, ^26^, ^27^, ^28^ Our estimates, therefore, reflect the contribution of HSV-2 infection to incident HIV infection only to the extent that the adjusted RRs accurately depict the magnitude of the effect of HSV-2 on HIV acquisition. Nonetheless, these estimates provide a starting point in understanding the potential contribution of HSV-2 infection to HIV acquisition globally, given the available data.

Finally, HSV-2 seems to have an additional cofactor effect on HIV transmissibility (ie, the biological association of HSV-2 infection on increasing HIV infectiousness),^4^ which was not included in our calculations and could increase the PAF of HIV acquisition attributable to HSV-2.^29^ Another consideration is that the classic PAF formula does not take into account the effect of HSV-2 infection on onward, secondary transmission of HIV.^30^ Because our PAF estimates were calculated for a single year (2016), we might have underestimated the contribution of HSV-2 infection to HIV acquisition over time. This work could be extended further with mathematical modelling of HSV-2 and HIV transmission at the population level to estimate how secondary infections resulting from the cofactor effect of HSV-2 infection on HIV acquisition further contribute to the PAF and to explore how changing epidemiology over time is reflected in the PAF. Mathematical modelling could also be used to explore the association between HIV and HSV-2 for more than one cofactor effect simultaneously.

Previously published PAF estimates^15^, ^16^, ^17^, ^18^, ^19^, ^20^ from some settings in Africa, which were derived using either the classic PAF formula^14^ or a statistical model (which uses a classic formula), varied according to the age and sex of the population for which the PAF was being estimated and the RR found by the study.^15^, ^16^, ^17^, ^18^, ^19^, ^20^ In a study in Mwanza (Tanzania), the PAF for HIV acquisition attributable to HSV-2 infection was calculated to be 59% using a statistical model, of which 46% was due to established HSV-2 infection and 13% to recently acquired HSV-2 infection.^15^ The PAF was found to be higher among men than women, with the lowest PAF found among women aged 15–24 years (12%, 95% CI −46 to 47) and the highest among men aged 25–54 years (76%, 43 to 90), although confidence intervals around these estimates were very wide.^16^ A slightly higher PAF, also obtained using a statistical model, was found for women (67%, 52–78) than for men (61%, 40–74) in a study in southwestern Uganda, with the PAF varying over time with the prevalence of HSV-2 infection.^17^ In a study in high-risk women in Mombasa (Kenya), the PAF was found to be 48% for established HSV-2 infection and 5% for recently acquired HSV-2 infection using a PAF formula based on HSV-2 status among incident HIV cases,^14^ but also in this case the PAF varied with the prevalence of HSV-2 infection over time.^18^ Using this same formula, the PAF was estimated to be 29% for established HSV-2 infection versus 2% for recently acquired HSV-2 infection among women in Harare (Zimbabwe), Durban, and Johannesburg (South Africa),^19^ and 50% for established infection versus 8% for recently acquired infection among women in Uganda and Zimbabwe.^20^

These estimates highlight the important link between HSV-2 and HIV infections and suggest that the global contribution of HSV-2 infection to HIV acquisition could be substantial. The link between HSV-2 and HIV infections has implications for both the management of people with known HSV-2 infection and for HSV-2 and HIV prevention efforts. For example, in settings or populations with high HIV incidence, individuals with symptoms suggestive of HSV-2 infection could benefit from HIV testing and more focused HIV prevention efforts, such as pre-exposure prophylaxis.^31^

New interventions against HSV-2, such as vaccines, new antivirals, or microbicides, have the potential to substantially reduce genital ulcer disease, which affects millions of people worldwide.^1^, ^32^, ^33^ In addition, our PAF estimates suggest that interventions directed against HSV-2 could also have an indirect effect on HIV incidence. This effect would enhance the public health value of HSV-2 prevention interventions. Even considering the uncertainty inherent in our analysis and in the absolute magnitude of the effect of HSV-2 infection on HIV acquisition, the fact that there were 1·5 million new HIV infections among individuals aged 15–49 years in 2016 and more than 400 million HSV-2 infections means that an indirect effect of HSV-2 prevention on HIV has the potential to prevent hundreds of thousands of HIV infections annually. In Africa, where HSV-2 prevalence is high in the heterosexual population, use of an effective intervention against HSV-2 would have the greatest potential effect on both HIV and HSV-2 infections. The effect would be less pronounced in regions such as the Eastern Mediterranean and Europe, where a higher proportion of HIV infections are related to injection drug use than in other regions.

Trials of daily suppressive antiviral therapy against HSV-2 did not show a reduction in HIV acquisition; however, it has been speculated that the antivirals evaluated did not sufficiently reduce the HSV-associated influx of immune cell targets for HIV for there to be an effect on HIV acquisition.^5^, ^8^, ^34^ Antivirals do not fully suppress viral shedding, leading to immune cell persistence,^9^, ^35^ and bioavailability of, and adherence to, aciclovir in the suppressive therapy trials were also cited as potential issues.^34^, ^36^, ^37^ New, more effective interventions could hold more promise for translating an effect on HSV-2 infection or shedding and disease into gains for the reduction of HIV incidence.^33^

The landscape for HIV prevention and treatment has transformed in the past decade, with expanded availability of HIV prevention interventions such as antiretroviral treatment, pre-exposure prophylaxis, and voluntary medical male circumcision.^38^ However, no intervention used alone is likely to be sufficient to achieve global goals to end the HIV epidemic, especially given challenges in uptake and adherence. Hence, combination measures are needed.^39^ A potential indirect effect of interventions against HSV-2 on HIV incidence will need to be evaluated in the context of existing HIV prevention interventions and for new interventions against HSV-2, particularly HSV-2 vaccines.^33^ Our estimates suggest that HSV-2 prevention measures could be an important additional tool in the fight against HIV.
