# Supplementary material for: Global and regional estimates of the contribution of herpes simplex virus type 2 infection to HIV incidence: a population attributable fraction analysis using published epidemiological data
Source: Lancet Infect Dis. 2020 Feb;20(2):240–9. doi: 10.1016/S1473-3099(19)30470-0 (PMC6990396; doi:10.1016/S1473-3099(19)30470-0)
Supplement: Supplementary appendix 2 [file mmc2.pdf]

THE LANCET Infectious Diseases

Supplementary webappendix

This webappendix formed part of the original submission and has been peer reviewed. We post it as supplied by the authors.

Supplement to: Looker KJ, Welton NJ, Sabin KM, et al. Global and regional estimates of the contribution of herpes simplex virus type 2 infection to HIV incidence: a population attributable fraction analysis using published epidemiological data. *Lancet Infect Dis* 2019; published online Nov 18. https://doi.org/10.1016/S1473-3099(19)30470-0.

Appendix: Global and regional estimates of the contribution of herpes simplex virus type 2 infection to HIV incidence: a population attributable fraction (PAF) analysis using published epidemiological data

Dr Katharine J. Looker, Professor Nicky J. Welton, Dr Keith M. Sabin, Dr Shona Dalal, Professor Peter Vickerman, Dr Katherine M. E. Turner, Professor Marie-Claude Boily and Dr Sami L. Gottlieb

FURTHER METHODS

References for FSW and MSM HSV-2 prevalence dataset

1. Baeten JM, Benki S, Chohan V, Lavreys L, McClelland RS, Mandaliya K, et al. Hormonal contraceptive use, herpes simplex virus infection, and risk of HIV-1 acquisition among Kenyan women. AIDS (London, England). 2007;21(13):1771-7.
2. Baeten JM, Hassan WM, Chohan V, Richardson BA, Mandaliya K, Ndinya-Achola JO, et al. Prospective study of correlates of vaginal Lactobacillus colonisation among high-risk HIV-1 seronegative women. Sexually transmitted infections. 2009;85(5):348-53.
3. Chohan V, Baeten JM, Benki S, Graham SM, Lavreys L, Mandaliya K, et al. A prospective study of risk factors for herpes simplex virus type 2 acquisition among high-risk HIV-1 seronegative women in Kenya. Sexually transmitted infections. 2009;85(7):489-92.
4. Bohl DD, Katz KA, Bernstein K, Wong E, Raymond HF, Klausner JD, et al. Prevalence and correlates of herpes simplex virus type-2 infection among men who have sex with men, san francisco, 2008. Sexually transmitted diseases. 2011;38(7):617-21.
5. Braunstein SL, Ingabire CM, Geubbels E, Vyankandondera J, Umulisa MM, Gahiro E, et al. High burden of prevalent and recently acquired HIV among female sex workers and female HIV voluntary testing center clients in Kigali, Rwanda. PloS one. 2011;6(9):e24321.
6. Braunstein SL, Ingabire CM, Kestelyn E, Uwizera AU, Mwamarangwe L, Ntirushwa J, et al. High human immunodeficiency virus incidence in a cohort of rwandan female sex workers. Sexually Transmitted Diseases. 2011;38(5):385-94.
7. Braunstein SL, van de Wijgert JH, Vyankandondera J, Kestelyn E, Ntirushwa J, Nash D. Risk factor detection as a metric of STARHS performance for HIV incidence surveillance among female sex workers in Kigali, Rwanda. Open AIDS Journal. 2012;6(SPEC.ISSUE 1):112-21.
8. Brown EL, Wald A, Hughes JP, Morrow RA, Krantz E, Mayer K, et al. High risk of human immunodeficiency virus in men who have sex with men with herpes simplex virus type 2 in the EXPLORE study. American journal of epidemiology. 2006;164(8):733-41.
9. Carcamo CP, Campos PE, Garcia PJ, Hughes JP, Garnett GP, Holmes KK. Prevalences of sexually transmitted infections in young adults and female sex workers in Peru: a national population-based survey. The Lancet infectious diseases. 2012;12(10):765-73.
10. Chen XS, Yin YP, Liang GJ, Gong XD, Li HS, Poumerol G, et al. Sexually transmitted infections among female sex workers in Yunnan, China. AIDS patient care and STDs. 2005;19(12):853-60.
11. Davies SC, Taylor JA, Sedyaningsih-Mamahit ER, Gunawan S, Cunningham AL, Mindel A. Prevalence and risk factors for herpes simplex virus type 2 antibodies among low- and high-risk populations in Indonesia. Sexually transmitted diseases. 2007;34(3):132-8.
12. Kane CT, Diawara S, Ndiaye HD, Diallo PA, Wade AS, Diallo AG, et al. Concentrated and linked epidemics of both HSV-2 and HIV-1/HIV-2 infections in Senegal: public health impacts of the spread of HIV. International journal of STD & AIDS. 2009;20(11):793-6.
13. Diawara S, Toure Kane C, Legoff J, Gaye AG, Mboup S, Belec L. Low seroprevalence of herpes simplex virus type 2 among pregnant women in Senegal. International journal of STD & AIDS. 2008;19(3):159-60.
14. Dolar N, Serdaroglu S, Yilmaz G, Ergin S. Seroprevalence of herpes simplex virus type 1 and type 2 in Turkey. Journal of the European Academy of Dermatology and Venereology : JEADV. 2006;20(10):1232-6.
15. Fox J, Taylor GP, Day S, Parry J, Ward H. How safe is safer sex? High levels of HSV-1 and HSV-2 in female sex workers in London. Epidemiology and infection. 2006;134(5):1114-9.
16. Hill C, McKinney E, Lowndes CM, Munro H, Murphy G, Parry JV, et al. Epidemiology of herpes simplex virus types 2 and 1 amongst men who have sex with men attending sexual health clinics in England and Wales: implications for HIV prevention and management. Euro surveillance : bulletin Europeen sur les maladies transmissibles = European communicable disease bulletin. 2009;14(47).
17. Holtz TH, Thienkrua W, McNicholl JM, Wimonsate W, Chaikummao S, Chonwattana W, et al. Prevalence of Treponema pallidum seropositivity and herpes simplex virus type 2 infection in a cohort of men who have sex with men, Bangkok, Thailand, 2006-2010. International journal of STD & AIDS. 2012;23(6):424-8.

18. Uma S, Balakrishnan P, Murugavel KG, Srikrishnan AK, Kumarasamy N, Cecelia JA, et al. Bacterial vaginosis in female sex workers in Chennai, India. Sexual health. 2005;2(4):261-2.
19. Jin F, Prestage GP, Mao L, Kippax SC, Pell CM, Donovan B, et al. Transmission of herpes simplex virus types 1 and 2 in a prospective cohort of HIV-negative gay men: the health in men study. The Journal of infectious diseases. 2006;194(5):561-70.
20. Jin F, Prestage GP, Imrie J, Kippax SC, Donovan B, Templeton DJ, et al. Anal sexually transmitted infections and risk of HIV infection in homosexual men. Journal of Acquired Immune Deficiency Syndromes. 2010;53(1):144-9.
21. Lama JR, Lucchetti A, Suarez L, Laguna-Torres VA, Guanira JV, Pun M, et al. Association of herpes simplex virus type 2 infection and syphilis with human immunodeficiency virus infection among men who have sex with men in Peru. The Journal of infectious diseases. 2006;194(10):1459-66.
22. Lupi O. Prevalence and risk factors for herpes simplex infection among patients at high risk for HIV infection in Brazil. International journal of dermatology. 2011;50(6):709-13.
23. Nagot N, Ouedraogo A, Defer MC, Vallo R, Mayaud P, Van de Perre P. Association between bacterial vaginosis and Herpes simplex virus type-2 infection: implications for HIV acquisition studies. Sexually transmitted infections. 2007;83(5):365-8.
24. O'Farrell N, Thuong NV, Nghia KV, Tram LT, Long NT. HSV-2 antibodies in female sex workers in Vietnam. International journal of STD & AIDS. 2006;17(11):755-8.
25. Nguyen VT, Khuu VN, Tran PH, Nguyen TL, Van CTB, Bui HD, et al. Impact of a community sexually transmitted infection/HIV intervention project on female sex workers in five border provinces of Vietnam. Sexually Transmitted Infections. 2007;83(5):376-82.
26. Papadogeorgaki H, Caroni C, Frangouli E, Flemetakis A, Katsambas A, Hadjivassiliou M. Prevalence of sexually transmitted infections in female sex workers in Athens, Greece - 2005. European journal of dermatology : EJD. 2006;16(6):662-5.
27. Rodrigues J, Grinsztejn B, Bastos FI, Velasque L, Luz PM, de Souza CT, et al. Seroprevalence and factors associated with herpes simplex virus type 2 among HIV-negative high-risk men who have sex with men from Rio de Janeiro, Brazil: a cross-sectional study. BMC infectious diseases. 2009;9:39.
28. Sanchez J, Lama JR, Kusunoki L, Manrique H, Goicochea P, Lucchetti A, et al. HIV-1, sexually transmitted infections, and sexual behavior trends among men who have sex with men in Lima, Peru. Journal of Acquired Immune Deficiency Syndromes. 2007;44(5):578-85.
29. Shin HS, Park JJ, Chu C, Song HJ, Cho KS, Lee JS, et al. Herpes simplex virus type 2 seroprevalence in Korea: rapid increase of HSV-2 seroprevalence in the 30s in the southern part. Journal of Korean medical science. 2007;22(6):957-62.
30. Wang H, Wang N, Chen RY, Sharp GB, Ma Y, Wang G, et al. Prevalence and predictors of herpes simplex virus type 2 infection among female sex workers in Yunnan Province, China. International journal of STD & AIDS. 2008;19(9):635-9.
31. Wang H, Reilly KH, Brown K, Jin X, Xu J, Ding G, et al. HIV incidence and associated risk factors among female sex workers in a high HIV-prevalence area of China. Sexually Transmitted Diseases. 2012;39(11):835-41.
32. Wang H, Brown KS, Wang G, Ding G, Zang C, Wang J, et al. Knowledge of HIV seropositivity is a predictor for initiation of illicit drug use: Incidence of drug use initiation among female sex workers in a high HIV-prevalence area of China. Drug and alcohol dependence. 2011;117(2-3):226-32.
33. Wang H, Wang N, Bi A, Wang G, Ding G, Jia M, et al. Application of cumulative odds logistic model on risk factors analysis for sexually transmitted infections among female sex workers in Kaiyuan city, Yunnan province, China. Sexually Transmitted Infections. 2009;85(4):290-5.
34. Wang HB, Smith K, Brown KS, Wang GX, Chang DF, Xu JJ, et al. Prevalence, incidence, and persistence of syphilis infection in female sex workers in a Chinese province. Epidemiology and Infection. 2011;139(9):1401-9.
35. Xu JJ, Smith MK, Chu J, Ding GW, Chang DF, Sharp GB, et al. Dynamics of the HIV epidemic in southern China: Sexual and drug-using behaviours among female sex workers and male clients in Yunnan. International Journal of STD and AIDS. 2012;23(9):670-5.
36. Yao Y, Yang F, Chu J, Siame G, Lim HJ, Jin X, et al. Associations between drug use and risk behaviours for HIV and sexually transmitted infections among female sex workers in Yunnan, China. International Journal of STD and AIDS. 2012;23(10):698-703.
37. Wang JJ, Zhu ZB, Yang X, Wu J, Wang HB, Feng L, et al. Herpes simplex virus type 2 risks in female sex workers in the China-Vietnam border county of Hekou. Biomedical and environmental sciences : BES. 2012;25(6):706-10.
38. Yang Y, Yao J, Gao M, Su H, Zhang T, He N. Herpes simplex virus type 2 infection among female sex workers in Shanghai, China. AIDS care. 2011;23 Suppl 1:37-44.

39. Yin YP, Chen SC, Wang HC, Wei WH, Wang QQ, Liang GJ, et al. Prevalence and risk factors of HSV-2 infection and HSV-2/HIV coinfection in men who have sex with men in China: a multisite cross-sectional study. Sexually transmitted diseases. 2012;39(5):354-8.
40. Yun H, Park J, Choi I, Kee M, Choi B, Kim S. Prevalence of human papillomavirus and herpes simplex virus type 2 infection in Korean commercial sex workers. Journal of microbiology and biotechnology. 2008;18(2):350-4.
41. Baltzer H, Chege D, Rebbapragada A, Wachihi C, Shin LYY, Kimani J, et al. Relative HIV resistance in kenyan sex workers is not due to an altered prevalence or mucosal immune impact of herpes simplex virus type 2 infection. Current HIV Research. 2009;7(5):504-7.
42. Barua P, Mahanta J, Medhi GK, Dale J, Paranjape RS, Thongamba G. Sexual activity as risk factor for hepatitis C virus (HCV) transmission among the female sex workers in Nagaland. Indian Journal of Medical Research. 2012;136(SUPPL.):30-5.
43. Bozicevic I, Lepej SZ, Rode OD, Grgic I, Jankovic P, Dominkovic Z, et al. Prevalence of HIV and sexually transmitted infections and patterns of recent HIV testing among men who have sex with men in Zagreb, Croatia. Sexually Transmitted Infections. 2012;88(7):539-44.
44. Bozicevic I, Rode OD, Lepej SZ, Johnston LG, Stulhofer A, Dominkovic Z, et al. Prevalence of sexually transmitted infections among men who have sex with men in Zagreb, Croatia. AIDS and behavior. 2009;13(2):303-9.
45. Chen S, Yin Y, Chen X, Wang H, Yu Y, Wei W, et al. Seropositivity and Risk Factors for Herpes Simplex Virus Type 2 Infection among Female Sex Workers in Guangxi, China. PloS one. 2013;8(7).
46. Perez-Brumer AG, Konda KA, Salvatierra HJ, Segura ER, Hall ER, Montano SM, et al. Prevalence of HIV, STIs, and Risk Behaviors in a Cross-Sectional Community- and Clinic-Based Sample of Men Who Have Sex with Men (MSM) in Lima, Peru. PloS one. 2013;8(4).
47. Clark JL, Konda KA, Segura ER, Salvatierra HJ, Leon SR, Hall ER, et al. Risk factors for the spread of HIV and other sexually transmitted infections among men who have sex with men infected with HIV in Lima, Peru. Sexually Transmitted Infections. 2008;84(6):449-54.
48. Creswell J, Guardado ME, Lee J, Nieto AI, Kim AA, Monterroso E, et al. HIV and STI control in El Salvador: Results from an integrated behavioural survey among men who have sex with men. Sexually Transmitted Infections. 2012;88(8):633-8.
49. Gul U, Kilic A, Sakizligil B, Aksaray S, Bilgili S, Demirel O, et al. Magnitude of sexually transmitted infections among female sex workers in Turkey. Journal of the European Academy of Dermatology and Venereology. 2008;22(9):1123-4.
50. Hawkes S, Collumbien M, Platt L, Lalji N, Rizvi N, Andreasen A, et al. HIV and other sexually transmitted infections among men, transgenders and women selling sex in two cities in Pakistan: A cross-sectional prevalence survey. Sexually Transmitted Infections. 2009;85(SUPPL. 2):ii8-ii16.
51. Hemalatha R, Hari Kumar R, Venkaiah K, Srinivasan K, Brahmam GNV. Prevalence of & knowledge, attitude & practices towards HIV & sexually transmitted infections (STIs) among female sex workers (FSWs) in Andhra Pradesh. Indian Journal of Medical Research. 2011;134(10):470-5.
52. Li D, Li S, Liu Y, Gao Y, Yu M, Yang X, et al. HIV incidence among men who have sex with men in Beijing: A prospective cohort study. BMJ Open. 2012;2(6).
53. Linhart Y, Shohat T, Amitai Z, Gefen D, Srugo I, Blumstein G, et al. Sexually transmitted infections among brothel-based sex workers in Tel-Aviv area, Israel: High prevalence of pharyngeal gonorrhoea. International Journal of STD and AIDS. 2008;19(10):656-9.
54. Mark HD, Sifakis F, Hylton JB, Celentano DD, Mackellar DA, Valleroy LA, et al. Sex with women as a risk factor for herpes simplex virus type 2 among young men who have sex with men in Baltimore. Sexually Transmitted Diseases. 2005;32(11):691-5.
55. Mishra S, Moses S, Hanumaiah PK, Washington R, Alary M, Ramesh BM, et al. Sex work, syphilis, and seeking treatment: an opportunity for intervention in HIV prevention programming in Karnataka, south India. Sexually Transmitted Diseases. 2009;36(3):157-64.
56. Pisani E, Purnomo H, Sutrisna A, Asy A, Zaw M, Tilman C, et al. Basing policy on evidence: Low HIV, STIs, and risk behaviour in Dili, East Timor argue for more focused interventions. Sexually Transmitted Infections. 2006;82(1):88-93.
57. Soto RJ, Ghee AE, Nunez CA, Mayorga R, Tapia KA, Astete SG, et al. Sentinel surveillance of sexually transmitted infections/HIV and risk behaviors in vulnerable populations in 5 Central American countries. Journal of Acquired Immune Deficiency Syndromes. 2007;46(1):101-11.
58. Van Griensven F, Thienkrua W, McNicholl J, Wimonsate W, Chaikummao S, Chonwattana W, et al. Evidence of an explosive epidemic of HIV infection in a cohort of men who have sex with men in Thailand. AIDS (London, England). 2013;27(5):825-32.

59. Vandenhoudt HM, Langat L, Menten J, Odongo F, Oswago S, Luttah G, et al. Prevalence of HIV and Other Sexually Transmitted Infections among Female Sex Workers in Kisumu, Western Kenya, 1997 and 2008. PloS one. 2013;8(1).
60. Vandepitte JM, Malele F, Kivuvu DM, Edidi S, Muwonga J, Lepira F, et al. HIV and other sexually transmitted infections among female sex workers in Kinshasa, Democratic Republic of Congo, in 2002. Sexually Transmitted Diseases. 2007;34(4):203-8.
61. Wayal S, Cowan F, Warner P, Copas A, Mabey D, Shahmanesh M. Contraceptive practices, sexual and reproductive health needs of HIV-positive and negative female sex workers in Goa, India. Sexually Transmitted Infections. 2011;87(1):58-64.
62. Xu JJ, Wang N, Lu L, Pu Y, Zhang GL, Wong M, et al. HIV and STIs in clients and female sex workers in mining regions of Gejiu city, China. Sexually Transmitted Diseases. 2008;35(6):558-65.
63. Znazen A, Frikha-Gargouri O, Berrajah L, Bellalouna S, Hakim H, Gueddana N, et al. Sexually transmitted infections among female sex workers in Tunisia: High prevalence of Chlamydia trachomatis. Sexually Transmitted Infections. 2010;86(7):500-5.
64. Holt BY, Effler P, Brady W, Friday J, Belay E, Parker K, et al. Planning STI/HIV prevention among refugees and mobile populations: situation assessment of Sudanese refugees. Disasters. 2003;27(1):1-15.
65. Jiang J, Cao N, Zhang J, Xia Q, Gong X, Xue H, et al. High prevalence of sexually transmitted diseases among men who have sex with men in Jiangsu Province, China. Sexually transmitted diseases. 2006;33(2):118-23.
66. Barnabas RV, Wasserheit JN, Huang Y, Janes H, Morrow R, Fuchs J, et al. Impact of herpes simplex virus type 2 on HIV-1 acquisition and progression in an HIV vaccine trial (the Step study). Journal of acquired immune deficiency syndromes (1999). 2011;57(3):238-44.

GATHER checklist

**Item no.**	**Item description**	**Location in manuscript**
**Objectives and funding**		
1	Objectives	Introduction
2	Funding	Role of the Funding Source
**Data inputs**		
3	Data collation	Methods
4	Inclusion and exclusion criteria	Methods
5	Data sources and characteristics	Methods, Supplementary Dataset and Appendix Further Methods
6	Potential sources of bias	Methods
7	Other data inputs	Methods
8	File of data inputs	Methods, Table 1, Appendix Supplementary Table 1, Supplementary Dataset and Appendix Further Methods
**Data analysis**		
9	Overview of method	Methods
10	Detailed description of methods	Methods
11	Selection of final model	Methods
12	Model evaluation and sensitivity analysis	N/A
13	Methods for calculating uncertainty	Methods
14	Model code	N/A
**Results and discussion**		
15	File of estimates	Figure 1 and Tables 2 and 3
16	Estimate uncertainty	Figure 1 and Tables 2 and 3
17	Results interpretation	Discussion
18	Limitations	Discussion

**Item number**	**Checklist item**
**Objectives and funding**	
1	Define the indicator(s), populations (including age, sex, and geographic entities), and time period(s) for which estimates were made.
2	List the funding sources for the work.
**Data inputs**	
For all data inputs from multiple sources that are synthesised as part of the study:	
3	Describe how the data were identified and how the data were accessed.
4	Specify the inclusion and exclusion criteria. Identify all ad-hoc exclusions.
5	Provide information about all included data sources and their main characteristics. For each data source used, report reference information or contact name/institution, population represented, data collection method, year(s) of data collection, sex and age range, diagnostic criteria or measurement method, and sample size, as relevant.
6	Identify and describe any categories of input data that have potentially important biases (eg, based on characteristics listed in item 5).
For data inputs that contribute to the analysis but were not synthesised as part of the study:	
7	Describe and give sources for any other data inputs.
For all data inputs:	
8	Provide all data inputs in a file format from which data can be efficiently extracted (eg, a spreadsheet rather than a PDF), including all relevant meta-data listed in item 5. For any data inputs that cannot be shared because of ethical or legal reasons, such as third-party ownership, provide a contact name or the name of the institution that retains the right to the data.
**Data analysis**	
9	Provide a conceptual overview of the data analysis method. A diagram may be helpful.
10	Provide a detailed description of all steps of the analysis, including mathematical formulae. This description should cover, as relevant, data cleaning, data pre-processing, data adjustments and weighting of data sources, and mathematical or statistical model(s).
11	Describe how candidate models were evaluated and how the final model(s) were selected.
12	Provide the results of an evaluation of model performance, if done, as well as the results of any relevant sensitivity analysis.
13	Describe methods of calculating uncertainty of the estimates. State which sources of uncertainty were, and were not, accounted for in the uncertainty analysis.
14	State how analytical or statistical source code used to generate estimates can be accessed.
**Results and discussion**	
15	Provide published estimates in a file format from which data can be efficiently extracted.
16	Report a quantitative measure of the uncertainty of the estimates (eg, uncertainty intervals).
17	Interpret results in light of existing evidence. If updating a previous set of estimates, describe the reasons for changes in estimates.
18	Discuss limitations of the estimates. Include a discussion of any modelling assumptions or data limitations that affect interpretation of the estimates.

Stevens GA, Alkema L, Black RE, Boerma JT, Collins GS, Ezzati M, et al. Guidelines for Accurate and Transparent Health Estimates Reporting: the GATHER statement. Lancet. 2016;388(10062):e19-e23.

Supplementary Table S1 Default parameter values, plausible ranges, and assumptions made on the annual number of incident HIV infections, the proportion of individuals with established HSV-2 infection and with recently-acquired HSV-2 infection, and the RR of HIV acquisition by HSV-2 status used to derive the PAF (%) for each WHO region, by age, sex and risk population

WHO region	Population		Annual number of incident HIV infections due to sexual transmission[1,2]			% of individuals with established HSV-2 infection			% of individuals with recently-acquired HSV-2 infection per year			RR of HIV acquisition for established HSV-2 infection			RR of HIV acquisition for recently-acquired HSV-2 infection		
			**Default value**	**Lower UB**	**Upper UB**	**Default value**	**Lower UI**	**Upper UI**	**Default value**	**Lower UB**	**Upper UB**	**Default value**	**Lower CI**	**Upper CI**	**Default value**	**Lower CI**	**Upper CI**
**Africa**	**Women and men**	15-49 years	932,000	792,000	1,082,000	31·5%	24·5%	39·8%	1·5%	--	--						
		15-24 years	419,000	356,000	486934	18·9%	13·9%	26·4%	2·1%	--	--	2·7[5]	2·2	3·4	4·7[5]	2·2	10·1
		25-49 years	512,000	435,000	595,000	40·6%	32·1%	49·4%	1·0%	--	--						
	**Women**	15-49 years	529,000	295,000	700,000	38·0%	30·0%	47·1%	1·6%	--	--						
		15-24 years	280,000	156,000	371,000	25·2%	18·6%	33·8%	2·6%	--	--	2·5[6]	1·8	3·4	7·2[6]	4·5	11·5
		25-49 years	249,000	139,000	330,000	47·1%	38·1%	56·5%	0·8%	--	--						
	**Men**	15-49 years	403,000	112,000	555,000	25·1%	19·1%	32·6%	1·4%	--	--						
		15-24 years	139,000	39,000	192,000	12·6%	9·3%	19·0%	1·6%	--	--	3·1[7]	2·2	4·3	4·7[10]	2·2	10·1
		25-49 years	264,000	73,000	363,000	34·1%	26·1%	42·4%	1·2%	--	--						
	**MSM**	15-49 years	66,000[3]	18,000[3]	91,000[3]	69·0%[4]	45·3%[11]	100·0%[11]	--	--	--	1·7[8]	1·4	2·1	NU	NU	NU
	**FSWs**	15-49 years	47,000[3]	26,000[3]	62,000[3]	81·4%	67·0%	90·4%	--	--	--	1·5[9]	0·8	2·7	NU	NU	NU
**Americas**	**Women and men**	15-49 years	140,000	120,000	163,000	14·4%	10·2%	20·5%	0·7%	--	--						
		15-24 years	49,000	42,000	57,000	6·0%	4·2%	9·3%	0·8%	--	--	2·7[5]	2·2	3·4	4·7[5]	2·2	10·1
		25-49 years	91,000	78,000	106,000	18·3%	13·0%	25·8%	0·7%	--	--						
	**Women**	15-49 years	40,000	33,000	47,000	18·4%	13·7%	24·6%	0·9%	--	--						
		15-24 years	17,000	15,000	20,000	7·8%	5·7%	11·3%	1·0%	--	--	2·5[6]	1·8	3·4	7·2[6]	4·5	11·5
		25-49 years	222,000	19,000	26,000	23·2%	17·3%	30·7%	0·8%	--	--						
	**Men**	15-49 years	100,000	81,000	124,000	10·3%	6·7%	16·3%	0·5%	--	--						
		15-24 years	32,000	26,000	40,000	4·3%	2·7%	7·3%	0·6%	--	--	3·1[7]	2·2	4·3	4·7[10]	2·2	10·1
		25-49 years	69,000	56,000	85,000	13·2%	8·6%	20·7%	0·5%	--	--						
	**MSM**	15-49 years	62,000[3]	50,000[3]	77,000[3]	37·3%	28·3%	47·2%	--	--	--	1·7[8]	1·4	2·1	NU	NU	NU
	**FSWs**	15-49 years	7,000[3]	6,000[3]	9,000[3]	83·7%	72·8%	90·7%	--	--	--	1·5[9]	0·8	2·7	NU	NU	NU
**Eastern Mediterranean**	**Women and men**	15-49 years	16,000	13,000	25,000	7·7%	4·7%[12]	13·4%[12]	0·3%	--	--						
		15-24 years	4,000	4,000	7,000	5·5%	3·2%[12]	10·6%[12]	0·5%	--	--	2·7[5]	2·2	3·4	4·7[5]	2·2	10·1
		25-49 years	11,000	9,000	18,000	9·0%	5·5%[12]	15·3%[12]	0·1%	--	--						
	**Women**	15-49 years	5,000	4,000	10,000	12·6%	8·0%[12]	19·8%[12]	0·4%	--	--						
		15-24 years	2,000	2,000	4,000	9·2%	5·8%[12]	15·2%[12]	0·8%	--	--	2·5[6]	1·8	3·4	7·2[6]	4·5	11·5
		25-49 years	3,000	3,000	6,000	14·5%	9·3%[12]	22·7%[12]	0·1%	--	--						
	**Men**	15-49 years	11,000	8,000	17,000	3·2%	1·8%[12]	6·5%[12]	0·1%	--	--	3·1[7]	2·2	4·3	4·7[10]	2·2	10·1
		15-24 years	2,000	2,000	4,000	2·0%	1·1%[12]	4·9%[12]	0·2%	--	--						

		25-49 years	8,000	6,000	13,000	3·9%	2·2%[12]	7·6%[12]	0·1%	--	--						
	**MSM**	15-49 years	8,000[3]	6,000[3]	13,000[3]	8·9%[4]	5·9%[11]	13·9%[11]	--	--	--	1·7[8]	1·4	2·1	NU	NU	NU
	**FSWs**	15-49 years	3,000[3]	2,000[3]	5,000[3]	17·1%	0·7%	85·7%	--	--	--	1·5[9]	0·8	2·7	NU	NU	NU
**Europe**	**Women and men**	15-49 years	126,000	120,000	133,000	7·1%	4·0%	12·5%	0·3%	--	--	2·7[5]	2·2	3·4	4·7[5]	2·2	10·1
		15-24 years	21,000	20,000	22,000	3·1%	1·9%	6·5%	0·4%	--	--						
		25-49 years	105,000	101,000	111,000	8·5%	4·8%	14·8%	0·3%	--	--						
	**Women**	15-49 years	51,000	46,000	56,000	9·8%	5·2%	17·8%	0·4%	--	--	2·5[6]	1·8	3·4	7·2[6]	4·5	11·5
		15-24 years	11,000	10,000	12,000	4·5%	2·7%	7·8%	0·5%	--	--						
		25-49 years	40,000	36,000	44,000	11·7%	6·1%	21·4%	0·3%	--	--						
	**Men**	15-49 years	75,000	71,000	79,000	4·3%	2·8%	7·3%	0·2%	--	--	3·1[7]	2·2	4·3	4·7[10]	2·2	10·1
		15-24 years	10,000	9,000	10,000	1·7%	1·1%	5·3%	0·2%	--	--						
		25-49 years	65,000	62,000	69,000	5·3%	3·4%	8·1%	0·2%	--	--						
	**MSM**	15-49 years	18,000[3]	17,000[3]	19,000[3]	10·3%	4·6%	21·7%	--	--	--	1·7[8]	1·4	2·1	NU	NU	NU
	**FSWs**	15-49 years	8,000[3]	7,000[3]	9,000[3]	66·6%	58·1%	74·1%	--	--	--	1·5[9]	0·8	2·7	NU	NU	NU
**South-East Asia**	**Women and men**	15-49 years	124,000	93,000	154,000	7·5%	3·6%	16·0%	0·4%	--	--	2·7[5]	2·2	3·4	4·7[5]	2·2	10·1
		15-24 years	61,000	46,000	76,000	3·6%	1·5%	8·6%	0·4%	--	--						
		25-49 years	63,000	47,000	78,000	9·6%	4·6%	19·8%	0·3%	--	--						
	**Women**	15-49 years	45,000	30,000	122,000	8·5%	4·8%	14·8%	0·4%	--	--	2·5[6]	1·8	3·4	7·2[6]	4·5	11·5
		15-24 years	26,000	17,000	70,000	3·6%	2·0%	6·4%	0·5%	--	--						
		25-49 years	20,000	13,000	53,000	11·0%	6·2%	19·1%	0·4%	--	--						
	**Men**	15-49 years	79,000	37,000	202,000	6·6%	2·4%	17·0%	0·3%	--	--	3·1[7]	2·2	4·3	4·7[10]	2·2	10·1
		15-24 years	36,000	17,000	91,000	3·7%	1·0%	10·6%	0·4%	--	--						
		25-49 years	43,000	20,000	111,000	8·2%	3·1%	20·4%	0·2%	--	--						
	**MSM**	15-49 years	27,000[3]	13,000[3]	69,000[3]	18·6%	16·2%	21·2%	--	--	--	1·7[8]	1·4	2·1	NU	NU	NU
	**FSWs**	15-49 years	8,000[3]	5,000[3]	22,000[3]	51·8%	37·4%	65·9%	--	--	--	1·5[9]	0·8	2·7	NU	NU	NU
**Western Pacific**	**Women and men**	15-49 years	74,000	35,000	133,000	8·0%	4·0%	16·6%	0·4%	--	--	2·7[5]	2·2	3·4	4·7[5]	2·2	10·1
		15-24 years	17,000	8,000	31,000	3·4%	1·6%	7·6%	0·4%	--	--						
		25-49 years	57,000	27,000	102,000	9·9%	5·0%	20·4%	0·3%	--	--						
	**Women**	15-49 years	18,000	15,000	22,000	11·9%	6·6%	20·9%	0·5%	--	--	2·5[6]	1·8	3·4	7·2[6]	4·5	11·5
		15-24 years	5,000	4,000	5,000	5·3%	2·7%	10·2%	0·6%	--	--						
		25-49 years	14,000	11,000	16,000	14·5%	8·1%	25·3%	0·5%	--	--						
	**Men**	15-49 years	55,000	44,000	65,000	4·3%	1·6%	12·5%	0·2%	--	--	3·1[7]	2·2	4·3	4·7[10]	2·2	10·1
		15-24 years	12,000	10,000	15,000	1·7%	0·6%	5·2%	0·2%	--	--						
		25-49 years	43,000	34,000	50,000	5·4%	2·0%	15·7%	0·2%	--	--						
	**MSM**	15-49 years	22,000[3]	18,000[3]	26,000[3]	9·5%	5·3%	16·6%	--	--	--	1·7[8]	1·4	2·1	NU	NU	NU
	**FSWs**	15-49 years	4,000[3]	3,000[3]	4,000[3]	58·3%	49·4%	66·7%	--	--	--	1·5[9]	0·8	2·7	NU	NU	NU

FSWs: female sex workers; MSM: men who have sex with men; UB: uncertainty bound; CI: confidence interval; NU: not used. Annual number of incident HIV infections due to sexual transmission shown to the nearest thousand. [1]Obtained by subtracting the estimated number of incident HIV infections in PWID from the total number of incident HIV infections, with the exception of incidence for FSWs and MSM. Numbers shown to

2 s. f. [2]Not all UB available: unavailable UB derived based on size of available UB for other estimates (as a proportion of the estimate). Numbers shown to 2 s. f. [3]Derived by applying the proportion of incident HIV infections estimated to be in FSWs and MSM to the total number of incident HIV infections. [4]Derived by applying the global mean ratio of prevalence in MSM to prevalence in general population men aged 15-49 years from other WHO regions to the prevalence in general population men 15-49 years in either Africa or Eastern Mediterranean, as applicable. [5]Estimate for general population women and men applied; all informing studies were from Africa. [6]Estimate for general population women applied; all informing studies were from Africa. [7]Estimate for general population men applied; all informing studies were from Africa. [8]Estimate for MSM applied; all informing studies were from outside Africa. [9]Estimate for FSWs applied; all informing studies but one were from Africa. [10]Estimate for general population women and men applied (from Africa), as only one informing study was available for men. Blank cell indicates data not available. [11]UB derived based on global mean size of UB for MSM (as a proportion of the estimate), truncating at 100%. [12]UB derived based on global mean size of UB for corresponding data (as a proportion of the estimate).

FURTHER RESULTS

Supplementary Table S2 Estimated global PAF (%) of incident sexually-acquired HIV infection and number of incident HIV infections in 2016 attributable to HSV-2 infection by WHO region, age, sex and risk population

Population		Incident HIV infection from sexual transmission attributable to HSV-2 infection (95%UI)		Population		Incident HIV infection from sexual transmission attributable to HSV-2 infection (95%UI)	
		**PAF (%)**	**Number**			**PAF (%)**	**Number**
**AFRICA GENERAL POPULATION**				**EUROPE GENERAL POPULATION**			
**Women**	**15-24 years**	35·2% (25·7%-45·7%)	98,603 (54,963-147,879)	**Women**	**15-24 years**	9·2% (5·6%-15·1%)	992 (590-1,606)
	**25-49 years**	43·1% (29·6%-54·7%)	107,208 (60,240-158,900)		**25-49 years**	16·5% (8·7%-28·5%)	6,597 (3,405-11,306)
	**15-49 years**	40·0% (27·9%-50·7%)	211,908 (117,207-318,816)		**15-49 years**	14·7% (7·8%-25·6%)	7,453 (4,039-13,046)
**Men**	**15-24 years**	24·5% (15·4%-36·1%)	34,031 (14,421-61,745)	**Men**	**15-24 years**	4·3% (2·0%-8·9%)	413 (195-867)
	**25-49 years**	43·2% (30·0%-55·8%)	113,906 (48,496-197,659)		**25-49 years**	10·7% (5·7%-18·0%)	6,955 (3,687-11,792)
	**15-49 years**	36·6% (24·5%-49·7%)	147,463 (63,349-256,103)		**15-49 years**	9·0% (4·8%-15·6%)	6,740 (3,523-11,837)
**Women and men**	**15-24 years**	28·5% (20·5%-37·8%)	119,706 (82,484-164,550)	**Women and men**	**15-24 years**	6·2% (3·4%-11·0%)	1,278 (705-2,235)
	**25-49 years**	42·1% (33·3%-51·7%)	215,853 (160,874-278,477)		**25-49 years**	13·4% (7·7%-21·9%)	14,143 (8,116-23,348)
**AFRICA KEY POPULATION ANALYSIS**				**EUROPE KEY POPULATION ANALYSIS**			
**FSWs**		28·9% (0%-60·2%)[1]	13,612 (0-30,418)[1,2]	**FSWs**		25·0% (0%-55·3%)[1]	1,994 (0-4,412)[1,2]
**MSM**		32·6% (3·3%-48·0%)[1]	21,460 (2,222-38,031)[1,2]	**MSM**		6·7% (2·5%-15·5%)[1]	1,212 (444-2,744)[1,2]
**AMERICAS GENERAL POPULATION**				**SOUTH-EAST ASIA GENERAL POPULATION**			
**Women**	**15-24 years**	15·2% (10·1%-22·3%)	2,625 (1,758-3,977)	**Women**	**15-24 years**	7·7% (4·7%-13·0%)	1,966 (0-4,818)
	**25-49 years**	28·6% (18·5%-39·1%)	6,365 (3,987-9,303)		**25-49 years**	16·1% (8·9%-26·4%)	3,200 (0-7,722)
	**15-49 years**	24·9% (15·5%-35·1%)	9,830 (6,028-14,755)		**15-49 years**	13·5% (7·5%-23·0%)	6,127 (0-15,278)
**Men**	**15-24 years**	9·9% (5·6%-17·3%)	3,148 (1,681-5,776)	**Men**	**15-24 years**	8·4% (3·0%-22·5%)	3,004 (0-10,046)
	**25-49 years**	22·9% (13·0%-36·0%)	15,693 (8,655-25,874)		**25-49 years**	15·1% (5·9%-34·8%)	6,546 (0-20,367)
	**15-49 years**	19·1% (10·9%-30·6%)	19,137 (10,496-32,230)		**15-49 years**	13·0% (5·2%-29·4%)	10,216 (0-33,057)
**Women and men**	**15-24 years**	11·6% (7·7%-17·4%)	5,667 (3,636-8,820)	**Women and men**	**15-24 years**	7·2% (3·6%-14·6%)	4,429 (2,089-9,028)
	**25-49 years**	25·2% (17·5%-34·5%)	22,860 (15,270-31,862)		**25-49 years**	14·8% (7·3%-26·5%)	9,353 (4,231-17,137)
**AMERICAS KEY POPULATION ANALYSIS**				**SOUTH-EAST ASIA KEY POPULATION ANALYSIS**			
**FSWs**		29·5% (0%-60·4%)[1]	2,125 (0-4,406)[1,2]	**FSWs**		20·6% (0-49·4%)[1]	1,669 (0-5,076)[1,2]
**MSM**		20·7% (12·6%-30·4%)[1]	12,824 (7,269-19,729)[1,2]	**MSM**		11·5% (6·8%-16·9%)[1]	3,114 (0-7,301)[1,2]
**EASTERN MEDITERRANEAN GENERAL POPULATION**				**WESTERN PACIFIC GENERAL POPULATION**			
**Women**	**15-24 years**	15·9% (10·2%-24·6%)	320 (114-593)	**Women**	**15-24 years**	10·6% (6·3%-17·5%)	493 (286-828)
	**25-49 years**	18·5% (10·3%-29·0%)	602 (214-1,153)		**25-49 years**	19·8% (11·1%-32·1%)	2,728 (1,487-4,418)
	**15-49 years**	17·6% (9·7%-27·9%)	924 (298-1,855)		**15-49 years**	17·4% (9·9%-28·5%)	3,197 (1,725-5,589)
**Men**	**15-24 years**	4·8% (2·3 %-9·7%)	116 (47-253)	**Men**	**15-24 years**	4·2% (1·7%-11·6%)	530 (217-1,523)
	**25-49 years**	7·9% (3·6%-15·8%)	643 (263-1,377)		**25-49 years**	10·9% (4·0%-25·6%)	4,665 (1,650-11,403)
	**15-49 years**	6·8% (3·2%-14·3%)	717 (278-1,624)		**15-49 years**	9·0% (3·4%-21·9%)	4,980 (1,878-12,213)

**Women and men**	**15-24 years**	10·1% (6·0%-17·3%)	448 (211-841)	**Women and men**	**15-24 years**	6·9% (3·6%-13·4%)	1,176 (359-2,690)
	**25-49 years**	13·6% (7·7%-21·0%)	1,550 (770-2,850)		**25-49 years**	15·3% (7·7%-28·3%)	8,675 (2,624-19,597)
**EASTERN MEDITERRANEAN KEY POPULATION ANALYSIS**				**WESTERN PACIFIC KEY POPULATION ANALYSIS**			
**FSWs**		7·9% (0%-45·0%)[1]	203 (0-1,233)[1,2]	**FSWs**		22·6% (0-51·7%)[1]	803 (0-1,880)[1,2]
**MSM**		5·9% (2·9%-10·8%)[1]	474 (189-968)[1,2]	**MSM**		6·2% (2·8%-11·5%)[1]	1,387 (570-2,611)[1,2]

UI: uncertainty interval; FSWs: female sex workers; MSM: men who have sex with men. Number of incident HIV infections attributable to HSV-2 infection calculated for each age and sex group separately; therefore estimates do not sum exactly across rows. Number of incident HIV infections attributable to HSV-2 infection not truncated to the nearest thousand as in main manuscript., however these estimates should not be interpreted as being as precise as this suggests. [1]Established HSV-2 infection considered only, and not additionally recently-acquired HSV-2 infection. [2]These numbers form a subset of the global numbers, and are not in addition. Estimates based on 2016 HIV incidence data, 2012 HSV-2 infection estimates, RR estimates from a literature review to 2017, and key population breakdown of HIV incidence for 2015.
